# Design and Performance Assessment of a High-Resolution Small-Animal PET System

**DOI:** 10.3390/bioengineering12101119

**Published:** 2025-10-19

**Authors:** Wei Liu, Peng Xi, Jiguo Liu, Xilong Xu, Zhaoheng Xie, Yanye Lu, Xiangxi Meng, Qiushi Ren

**Affiliations:** 1 College of Future Technology, Peking University, Beijing 100871, China; liuw87@pku.edu.cn (W.L.); xpeng@pku.edu.cn (P.X.); 2 Shandong Madic Technology Co., Ltd., Linyi 276100, China; liujg@madiclab.com (J.L.); xuxl@madiclab.com (X.X.); 3 Institute of Medical Technology, Peking University Health Science Center, Peking University, Beijing 100191, China; xiezhaoheng@pku.edu.cn (Z.X.); yanye.lu@pku.edu.cn (Y.L.); 4 Department of Nuclear Medicine, Peking University Cancer Hospital, Beijing 100142, China; mengxiangxi@pku.edu.cn; 5 Shenzhen Bay Laboratory, Shenzhen 518132, China

**Keywords:** small-animal PET, LYSO, SiPM, performance evaluation

## Abstract

This work reports the performance evaluation of a newly developed small-animal positron emission tomography (PET) system based on lutetium-yttrium oxyorthosilicate (LYSO) crystals and multi-pixel photon counter (MPPC). Performance was evaluated, including spatial resolution, system sensitivity, energy resolution, scatter fraction (SF), noise–equivalent count rate (NECR), micro-Derenzo phantom imaging, and in vivo imaging of mice and rats. The system achieved a tangential spatial resolution of 0.9 mm in the axial direction at a quarter axial offset using the three-dimensional ordered-subsets expectation maximization (3D OSEM) reconstruction algorithm. The peak sensitivity was 8.74% within a 200–750 keV energy window, with an average energy resolution of 12.5%. Scatter fractions were 12.9% and 30.0% for mouse- and rat-like phantoms, respectively. The NECR reached 878.7 kcps at 57.6 MBq for the mouse phantom and 421.4 kcps at 63.2 MBq for the rat phantom. High-resolution phantom and in vivo images confirmed the system’s capability for quantitative, high-sensitivity small-animal imaging, demonstrating its potential for preclinical molecular imaging studies.

## 1. Introduction

Positron emission tomography (PET) is an essential imaging modality in many clinical fields, including oncology, neurology, and cardiology [1,2,3]. PET detects gamma emissions from radiopharmaceuticals and non-invasively reveals in vivo biological processes and physiological activities [4]. In preclinical research, small-animal imaging has become a cornerstone for various biological research fields [5]. Among various small-animal imaging modalities, PET plays a crucial role due to its high resolution, high sensitivity, and quantitative capability [6]. These strengths enable small-animal PET to effectively characterize tumors, brain structures, cardiovascular diseases, and other conditions [7], thereby accelerating translational research.

Continuous improvements in detector materials and components have significantly enhanced the performance of PET systems. In particular, silicon photomultipliers (SiPMs), also known as multi-pixel photon counters (MPPCs, the trade name used by Hamamatsu Photonics [8]), have become widely used in PET systems. SiPM or MPPC combines high gain, compact size, low operating voltage, and insensitivity to magnetic fields, making them well-suited for integration in modern PET systems [9,10]. For the scintillator, LYSO has become one of the most widely used materials in modern PET scanners due to its high light output, high stopping power, and short decay time [11]. Monolithic and semimonolithic crystals [12,13] are advantageous in providing higher sensitivity and depth-of-interaction (DOI) information, but they require complex optical modeling and advanced position decoding, which considerably increases system complexity. However, in this work, we adopted pixelated LYSO arrays as a practical trade-off, since they provide straightforward event positioning, easier integration with SiPM-based readout, and robust system stability, explaining their widespread adoption in both research prototypes and commercial scanners. The combination of LYSO scintillators with SiPM has demonstrated excellent timing, energy, and spatial resolution, as well as high system sensitivity [14].

Recently, we developed a new high-resolution small animal PET system, PKU-PET-III, as the successor to our previous-generation scanner (PKU-PET-II [15]). In the earlier system, we adopted a one-to-one crystal-to-SiPM coupling design that enhanced spatial resolution and sensitivity. However, this approach constrained the crystal size to match the SiPM dimensions, limiting the implementation of smaller crystals to further improve resolution. In PKU-PET-III, design optimizations allowed the use of smaller crystals, thereby overcoming this limitation. The scanner adopts a half-coupled crystal design, with LYSO positioning determined by an Anger logic circuit. Additionally, the PET detector functions without the need for a dedicated cooling system, simplifying system integration and reducing hardware complexity

This paper presents a detailed description of the system design, followed by a quantitative evaluation of the PET system, including sensitivity, spatial resolution, energy resolution, scatter fraction (SF), count losses, random coincidence measurements, and image quality evaluation using both phantom and in vivo experiments. Most of the experiments were performed in accordance with the relevant NEMA NU 4-2008 guidelines [16], where applicable.

## 2. Materials and Methods

### 2.1. PET System Description

**Figure 1:**
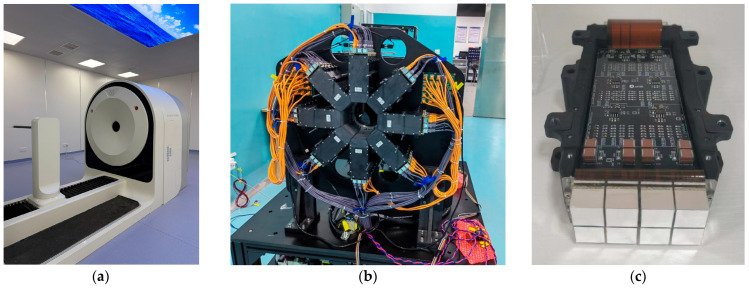
External appearance of the PKU-PET-III system (**a**), PET detector ring (**b**), and detector module (**c**).

The PET imaging system (as shown in Figure 1a) is based on LYSO scintillation crystals optically coupled with MPPC arrays (Hamamatsu Photonics K.K., Hamamatsu City, Japan; model S14160-4075HS). As shown in Figure 1b, the scanner comprises 32 detector modules arranged in four axial rings with eight modules per ring, resulting in a 129 mm ring diameter. The scanner provides a transaxial field of view (FOV) of 81 mm and an axial FOV of 122 mm. Each detector module contains 8 blocks, and each block consists of an 8 × 8 array of LYSO crystals optically coupled via a light guide to a 4 × 4 MPPC array, as shown in Figure 1b. In the system geometry, lines of response (LORs) were not formed between radially adjacent modules or between detector modules located in the same axial plane. The individual crystal elements measure 1.457 × 1.457 × 12 mm^3^, and each MPPC pixel has dimensions of 3 × 3 mm^2^. The main structural parameters of the PET system are summarized in Table 1, together with those of the previous PKU-PET-II system for comparison.

### 2.2. Performance Test

#### 2.2.1. Energy Resolution

The NEMA NU 4-2008 standard does not specify a procedure for measuring energy resolution. Therefore, we evaluated this parameter according to the conventional definition, where energy resolution is calculated as the ratio of the full width at half maximum (FWHM) of the photopeak to its energy (511 keV) [17]. A ^22^Na point source with an activity of 0.37 MBq was positioned at the center of the FOV, in both axial and transaxial directions. Singles events were acquired for 30 min to generate two-dimensional (2D) histograms [18] for each crystal without applying an energy window. The lower and upper thresholds of 250, 300, 350, and 750 keV were defined relative to this reference, assuming near-linearity of the SiPM and front-end electronics response within the 100–750 keV range. Bench tests with additional γ sources confirmed that any residual nonlinearity is negligible for the energy windows used in this study.

The energy spectrum of each crystal was analyzed to create a lookup table (LUT) [19] for all 32 detector modules. The energy resolution of each crystal was calculated from its spectrum, and the overall system energy resolution was obtained by averaging across all crystals, as expressed in Equations (1) and (2).

(1) $$E_{k}=\frac{{FWHM}_{k}}{e_{k}}\times 100\%\;,$$

(2) $$E_{sys}=\frac{\sum_{k=1}^{n}E_{k}}{n},$$

where ${FWHM}_{k}$ is the full width at half maximum of the energy peak for the kth crystal detector element; $e_{k}$ is the photopeak value; $E_{k}$ is the energy resolution of the kth crystal detector element; $E_{sys}$ is the overall system energy resolution; and *n* is the total number of crystals.

#### 2.2.2. Spatial Resolution

To evaluate the spatial resolution of the PET system, a ^22^Na point source with an initial activity of 1.457 MBq measuring 0.2 mm in diameter and embedded within a 1 cm^3^ acrylic cube was used. A component-based normalization method was applied to correct variations in detector efficiency. Following the guidelines of the NEMA NU 4-2008 standard, the point source was placed both at the center of the axial FOV and one-quarter of the axial offset for testing. According to the standard, point sources are positioned at 5, 10, 15, 20, and 25 mm radial offsets along the transaxial direction. However, given the relatively large transverse FOV of the system, extra measurements were conducted at the transaxial center and at 30 and 35, and 40 mm offsets to cover the system’s large transverse FOV. For each position, at least 100k prompt events were collected with a 200–700 keV energy window and a 6 ns coincidence timing window.

To assess the spatial resolution of the PET system, images were reconstructed using the 3D OSEM algorithm with five subsets and four iterations. Spatial resolution was quantified as the FWHM of reconstructed point-source images in the tangential, radial, and axial directions. Peak values were determined by parabolic fitting of the maximum point and its two neighbors, while FWHM values were computed via linear interpolation between adjacent points at half-maximum intensity. The final resolution was calculated by multiplying the interpolated width by the image isotropic voxel size of 0.486 mm.

#### 2.2.3. Micro-Derenzo Phantom

**Figure 2:**
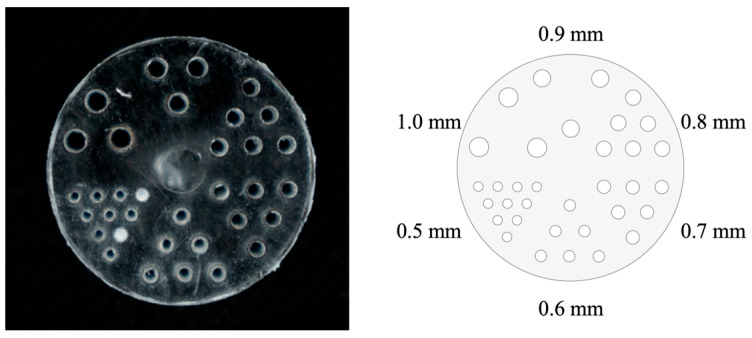
Micro–Derenzo phantom (rods’ diameters range from 0.5 to 1.0 mm).

The micro-Derenzo phantom is widely used to evaluate the spatial resolution of PET systems, offering an intuitive visualization of imaging performance [20]. As shown in Figure 2, the phantom used in this study contains six groups of holes with the following diameters: 0.5, 0.6, 0.7, 0.8, 0.9, and 1.0 mm. This configuration was selected to evaluate the upper limit of the system spatial resolution when reconstructed with the 3D OSEM algorithm.

The phantom was positioned at the center of the animal bed, and imaging was conducted with an initial activity of 2.78 MBq of fluorine-18 fluorodeoxyglucose (^18^F-FDG). Data acquisition lasted 20 min, and the acquired data were reconstructed using the 3D OSEM algorithm.

#### 2.2.4. Sensitivity

A ^22^Na point source with an initial activity of 0.37 MBq was used to evaluate the system sensitivity. The source was first positioned at the center of the axial FOV and then moved stepwise by 2.5 mm along the axial direction to cover the entire axial FOV. At each position, 10,000 true events were acquired, and the acquisition time (T_acq_) was noted. Background true event rates (*R_B,i_*) were measured without the radioactive source for 3 min and applied to correct the counting rates in each measurement, as described by Equation (3).

(3) $$S_{i}=\frac{R_{i}-R_{B,i}}{A_{cal}}$$

The final sensitivity was calculated using Equation (4).

(4) $$S_{A,i}=\frac{S_{i}}{0.906}\times 100$$

where $S_{i}$ represents the sensitivity of slice *i*, $R_{i}$ is the count rate (counts per second) for slice *i*, $R_{B,i}$ denotes the background count rate of slice *i*, and $A_{cal}$ is the activity of the source. The branching ratio of the ^22^Na point source is 0.906. Measurements were performed with three energy windows (200–750 keV, 300–750 keV, 350–750 keV) and a fixed 6 ns coincidence window. The 200–750 keV setting was applied during early system characterization to ensure sufficient count statistics across all detector modules, while the narrower windows of 300–750 keV and 350–750 keV were applied in routine imaging studies to reduce scatter acceptance and thereby achieve higher image quality and quantitative accuracy.

#### 2.2.5. Scatter Fraction and Count Rate Performance

**Figure 3:**
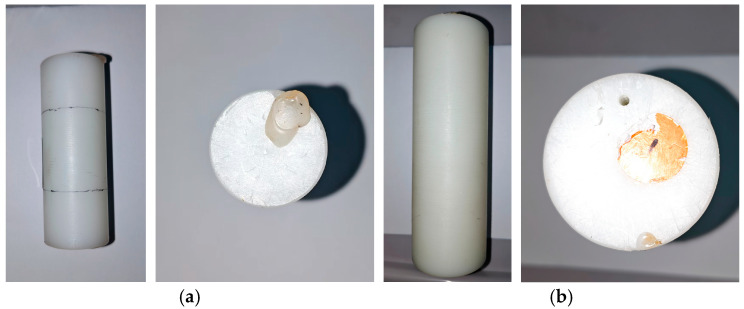
Mouse phantom (**a**) and rat phantom (**b**).

The NEMA NU 4-2008 standard defines three cylindrical phantoms to simulate mice, rats, and monkeys, with dimensions of 70 mm × Ø 25 mm, 150 mm × Ø 50 mm, and 400 mm × Ø 100 mm (length × diameter), respectively. As the current PET system is not compatible with the monkey phantom, testing was performed exclusively with the mouse and rat phantoms, as shown in Figure 3.

To ensure accurate assessment of the peak true coincidence rate and NECR, each phantom was filled with a sufficiently high initial activity of ^18^F-FDG to induce measurable detector dead time effects. Insufficient activity levels would fail to adequately characterize the system’s intrinsic performance. In this study, initial ^18^F-FDG activities of 57.6 MBq and 63.2 MBq were applied to the mouse and rat phantoms, respectively.

Data acquisitions were performed in 1 min intervals every 30 min until the true event losses were below 1.0% or the ratio of random-to-true event rates dropped below 1.0%. List-mode data were reformatted into sinograms using the single-slice rebinning (SSRB) method to enable a faster reconstruction speed [21]. Additionally, intrinsic background counts were collected for 3 min with no radioactivity present in the phantoms. Random coincidence events were estimated using the delayed window method.

Data processing was conducted following the NEMA NU 4-2008 standard. The scatter fraction and NECR were calculated using the following equations.

(5) $$R_{scatter}=R_{tot}-R_{true}-R_{random}-R_{int}$$

(6) $$SF=\frac{R_{scatter}}{R_{true}+R_{scatter}}$$

(7) $$R_{NEC}=\frac{R_{true}^{2}}{R_{tot}}$$

where $R_{scatter}$, $R_{tot}$, $R_{true}$, $R_{random}$, and $R_{int}$ represent the scatter, total, true, random, and intrinsic count rates, respectively, while $R_{NEC}$ denotes the noise-equivalent count rate.

#### 2.2.6. Image Quality

Image quality was evaluated using the NEMA NU 2-2008 image quality phantom, which consists of a hot region filled with ^18^F-FDG, five rods with diameters of 1, 2, 3, 4, and 5 mm connected to the hot region. In addition, two cylindrical cold chambers (8 mm in diameter, 15 mm in length), one filled with water and the other with air, were included to evaluate the accuracy corrections.

Data acquisition commenced when the total activity within the phantom decayed to 3.7 MBq. A 20 min static scan was performed, and image reconstruction was conducted using the maximum likelihood expectation maximization (MLEM) algorithm with 20 iterations, a matrix size of 166 × 166 × 252, and an isotropic voxel size of 0.486 mm.

Three quantitative image quality metrics were evaluated: uniformity, recovery coefficients (RC), and accuracy of corrections. Uniformity was obtained with a cylindrical volume of interest (VOI) (10 mm in length, 22.5 mm in diameter) was placed at the center of the uniform background region. For the RC, five circular ROIs (diameter twice that of the rod) were drawn on transverse slices. Record the coordinates of the maximum pixel value in each ROI and draw contour lines along the axis of the rod. The average pixel value measured by each contour, divided by the average activity concentration of the uniform area, is used to determine the recovery coefficient of each rod. The calculation formula of the RC is as follows:

(8) $$\%{STD}_{RC}=100\times \sqrt{{\left(\frac{{STD}_{lineprofile}}{{Mean}_{lineprofile}}\right)}^{2}+{\left(\frac{{STD}_{background}}{{Mean}_{background}}\right)}^{2}}$$

where $\%{STD}_{RC}$ represents the percentage standard deviation of the recovery coefficient. ${STD}_{lineprofile}$ and ${Mean}_{lineprofile}$ denote the standard deviation and mean activity concentration derived from the line profiles, while ${STD}_{background}$ and ${Mean}_{background}$ correspond to the standard deviation and mean activity concentration obtained from the uniformity region.

The accuracy of corrections is calculated by drawing cylindrical VOIs with a length of 7.5 mm and a diameter of 4 mm separately in the water-filled and air-filled areas. The calculation formula for the spill-over ratio is as follows:

(9) $$SOR=\frac{{Mean}_{coldregion}}{{Mean}_{hotregion}}$$

where SOR denotes the spill-over ratio. ${Mean}_{coldregion}$ refers to the mean activity concentration of the VOI placed at the center of the water-or air-filled cold region, while ${Mean}_{hotregion}$ represents the mean activity concentration of the VOI within the uniform background region.

#### 2.2.7. Animal Image Studies

All animal studies were conducted in compliance with institutional guidelines and approved by the Shenzhen Bay Laboratory Ethics Committee (approval no. AFLQ202201). The animal studies included both rat and mouse experiments. The rat measured 25 cm in length and weighed 140.2 g, while the mouse measured 12 cm in length and weighed 45 g. The rat and mouse were intravenously injected with 0.56 MBq and 0.329 MBq of ^18^F-FDG, respectively. After an uptake period of 50 min, the imaging duration was 30 min for the mouse and 15 min for the rat, respectively. Image reconstruction was conducted using the 3D-OSEM algorithm with 24 and 14 iterations, respectively. Throughout the acquisition, gas anesthesia was administered to maintain immobilization of the animals.

## 3. Results

### 3.1. Energy Resolution

**Figure 4:**
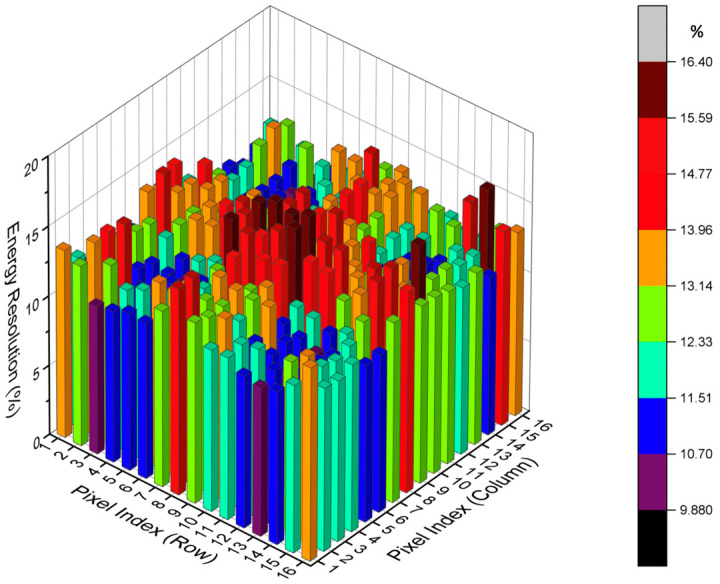
Histograms of the energy resolution for 4 crystal blocks (16 × 16).

The PET system showed an average energy resolution of 12.5%, ranging from 9.4% to 23.2%. The energy resolution distributions of 4 adjacent detector blocks selected from the total 256 blocks are shown in Figure 4.

### 3.2. Spatial Resolution

**Figure 5:**
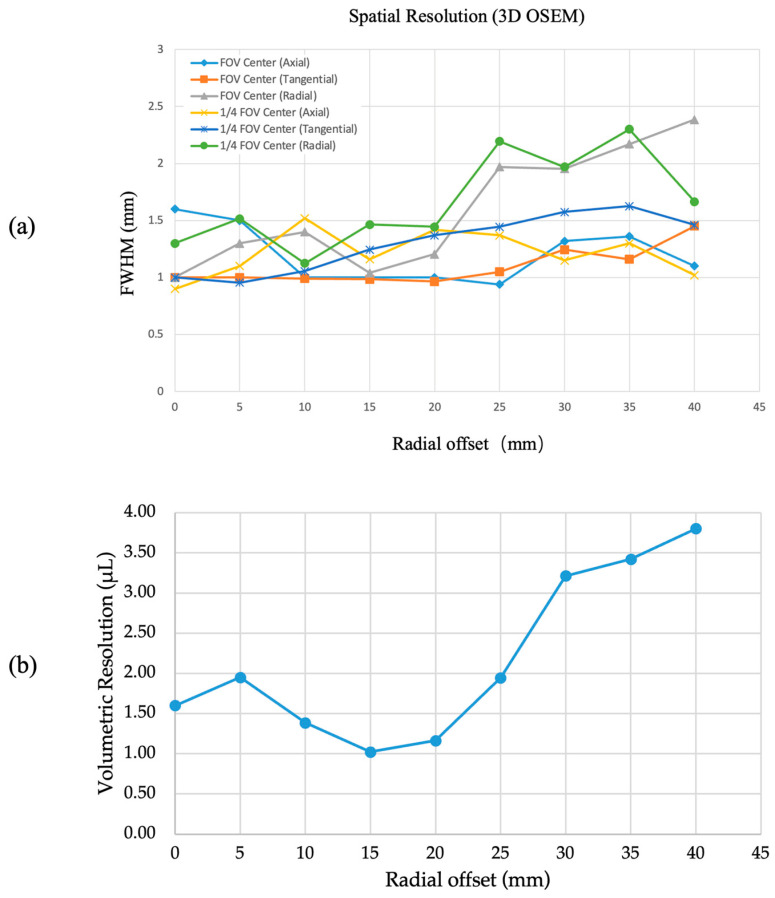
(**a**) Spatial resolution measured at the axial FOV center and at a quarter axial offset, reconstructed using the 3D OSEM algorithm. (**b**) Corresponding volumetric resolution.

Figure 5 shows the spatial resolution measured in the tangential, radial, and axial directions at multiple radial offsets, both at the center of the axial FOV and at a quarter axial offset. The best resolution was achieved in the axial direction at a quarter axial offset, reaching 0.9 mm (FWHM) with the 3D OSEM reconstruction algorithm. Across radial offsets from the center to 40 mm, the spatial resolution ranged from 0.9 mm to 2.39 mm, with improved uniformity observed in the axial and tangential directions, where it ranged from 0.9 mm to 1.63 mm. In addition, the corresponding volumetric resolution, calculated as the product of the radial, tangential, and axial FWHM values, is presented in Figure 5b. At the CFOV, the volumetric resolution of PKU-PET-III was approximately 1.60 mm^3^. At 15 mm radial offset, the volumetric resolution reached 1.02 mm^3^, representing the best value observed across the FOV.

### 3.3. Micro Derenzo Phantom

**Figure 6:**
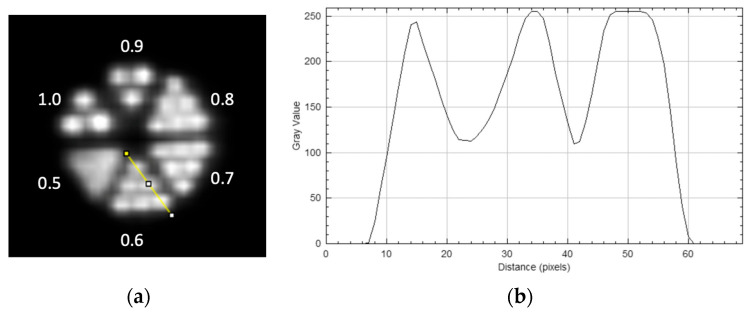
PET image (**a**) of the micro-Derenzo phantom reconstructed with the 3D OSEM algorithm (12 subsets, 5 iterations) and line profile (**b**) at the 0.6 mm rods corresponding to the location indicated by the short line.

As shown in Figure 6, the micro-Derenzo phantom image reconstructed with the 3D OSEM algorithm exhibited high spatial resolution. Rods as small as 0.6 mm were visualized (modulation contrast ≈ 39%), while clear separation is observed at 0.9 mm. The limited visual improvement among the 0.6–0.8 mm groups may be related to the system’s modulation transfer function near the resolvable limit, as well as partial-volume effects, reconstruction regularization, and sampling/voxel-size constraints at sub-millimeter scales. This observation is consistent with the point-source resolution measurements and suggests that the system is capable of sub-millimeter imaging under practical reconstruction settings.

### 3.4. Sensitivity

**Figure 7:**
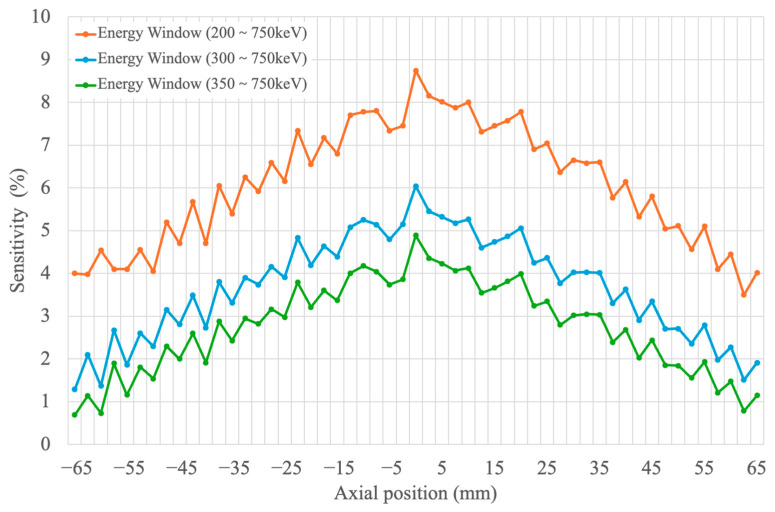
Axial sensitivity profiles of the small-animal PET system measured with energy windows of 200–750 keV, 300–750 keV, and 350–750 keV. At the extreme axial edges, the sensitivity did not fully drop to zero due to residual scatter, random events, and intrinsic ^176^Lu background, but this effect was negligible for the peak sensitivity evaluation.

Figure 7 shows the system sensitivity evaluated with three energy windows. At the axial center, the peak sensitivities for the 200–750 keV, 300–750 keV, and 350–750 keV windows were 8.74%, 6.04%, and 4.89%, respectively. These results highlight the strong dependence of system sensitivity on energy window selection.

### 3.5. Scatter Fraction and Count Rate Performance

**Figure 8:**
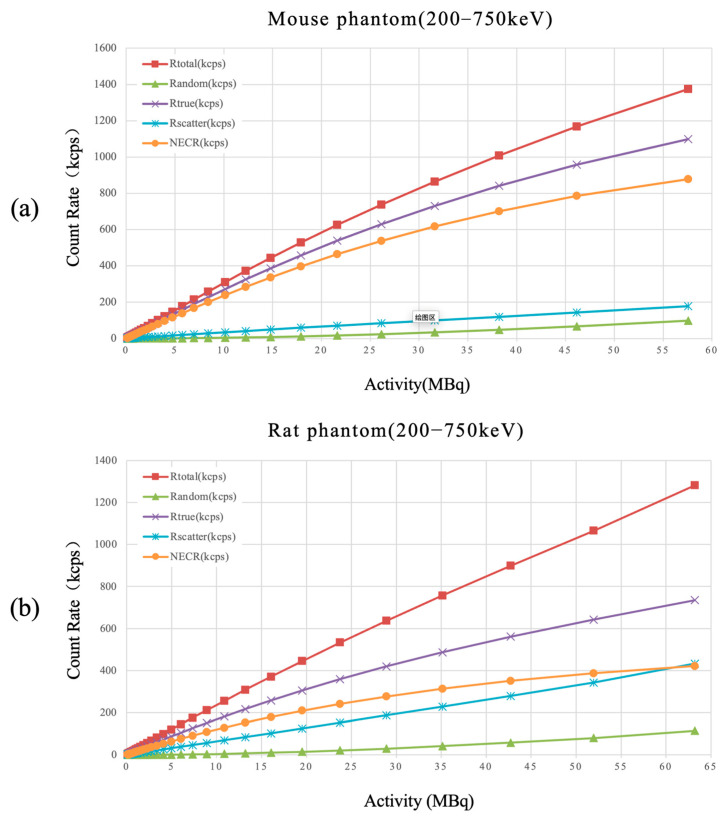
Count rates for total, true, random, scattered events, and NECR, plotted against total activity (MBq) for (**a**) mouse phantom and (**b**) rat phantom.

The scatter fractions for the mouse and rat phantoms were 12.9% and 30%, respectively. The NECR reached 878.7 kcps at 57.6 MBq for the mouse phantom and 421.4 kcps at 63.2 MBq for the rat phantom. The measured total, true, random, and scattered event rates are presented in Figure 8. Within the investigated activity range, the count rates and NECR increased monotonically without showing saturation effects. This is mainly because the activity levels used in our experiments did not reach the region where the acquisition electronics become saturated. In practice, these activity levels already cover the typical injected dose ranges used in preclinical imaging, suggesting that the system can provide stable performance within the relevant operational range.

### 3.6. Image Quality

**Figure 9:**
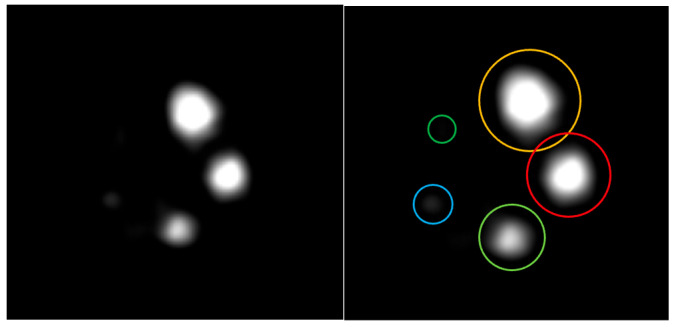
The image of IQ phantom.

**Figure 10:**
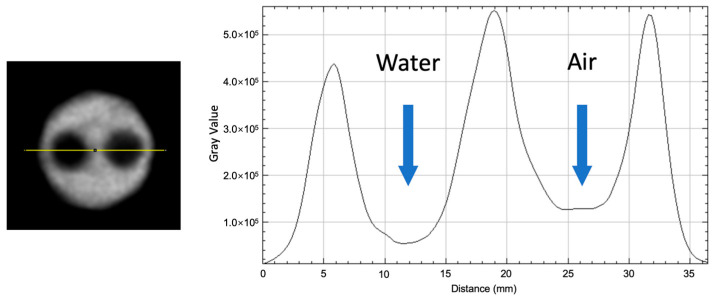
Image quality phantom. Cross-section (**left**) and activity line profile (**right**) along the water-filled region and the air-filled region. Peaks correspond to rod regions, while valleys correspond to water- and air-filled backgrounds, respectively. The *Y*-axis denotes relative voxel intensity (gray values).

**Figure 11:**
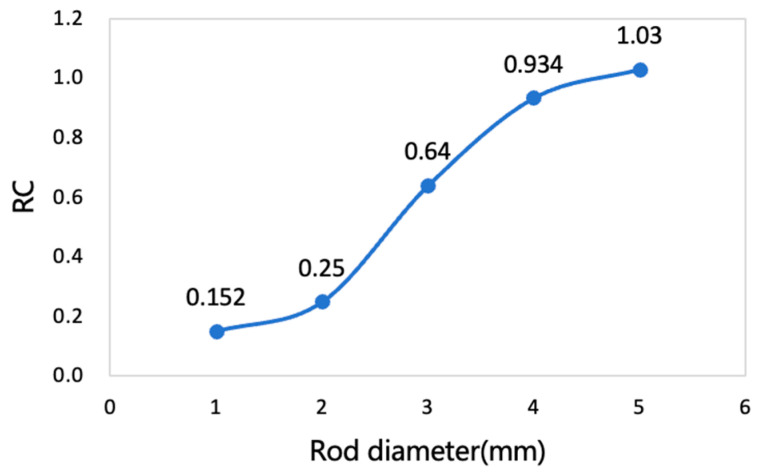
The RCs of five rods in the defined ROIs.

The image of the IQ phantom is shown in Figure 9. A cross-section of the cold regions in the image quality phantom, along with the activity profile in the cold regions, is also presented in Figure 10. The average, maximum, minimum activity concentration, and the %STD in the uniform area are shown in Table 2. RCs for five rods of different sizes are shown in Figure 11. The SOR for the water-filled chamber and the air-filled chamber are shown in Table 3.

### 3.7. Animal Image Studies

**Figure 12:**
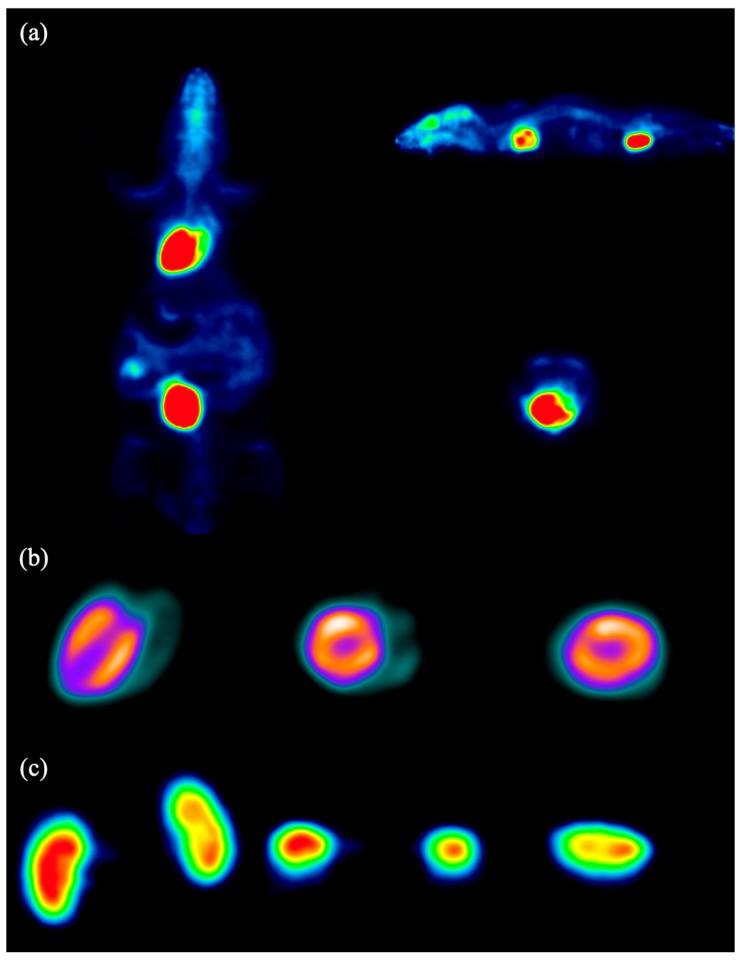
PET images of the mouse body (**a**), heart (**b**), and kidney (**c**). The colors represent relative radiotracer uptake, with warmer colors (red–yellow) indicating higher activity and cooler colors (green–blue) indicating lower activity.

**Figure 13:**
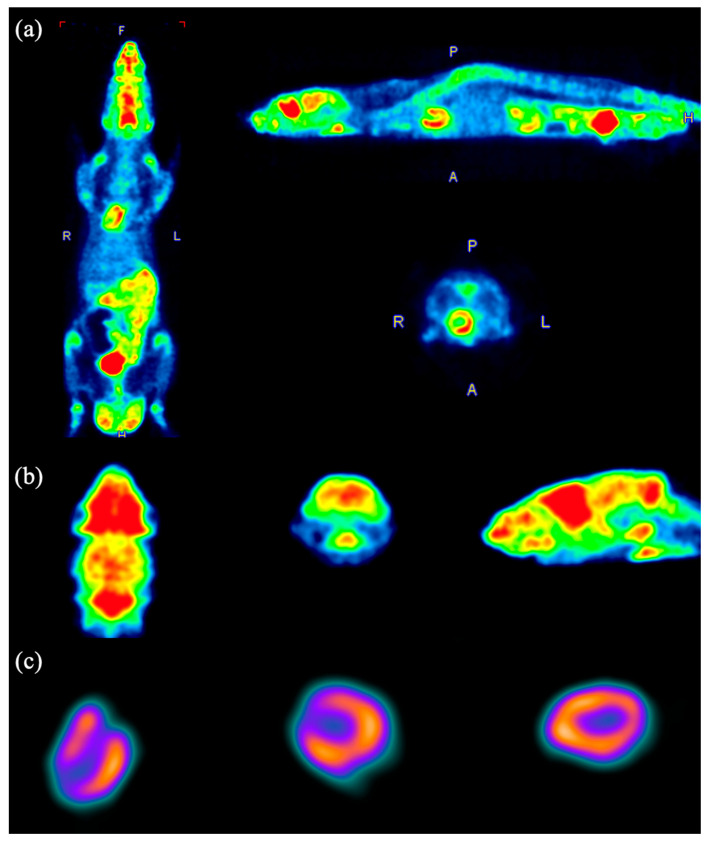
PET images of the rat body (**a**), brain (**b**), and heart (**c**). Colors follow the same convention in Figure 12, where warmer tones indicate higher activity and cooler tones indicate lower activity.

Figure 12 and Figure 13 show the PET images of a mouse and a rat, respectively, both injected with ^18^F-FDG. For the mouse, images corresponding to the whole body (a), heart (b), and kidneys (c) are displayed, while for the rat, images of the body (a), brain (b), and heart (c) are provided.

## 4. Discussion

In this study, we evaluated the performance of a newly developed small-animal PET in accordance with the relevant guidelines of NEMA NU 4-2008. The system features a low-cost yet high-performance design, providing a robust foundation for integration into a multi-modality imaging platform.

Spatial resolution for PKU-PET-II (FBP) at CFOV and 5 mm offset was 1.70 ± 0.10, 1.68 ± 0.07, and 1.92 ± 0.05 mm (radial, tangential, axial) at FWHM. For PKU-PET-III reconstructed with OSEM, the corresponding values were 1.3, 1.0, and 1.5 mm. As OSEM and FBP are known to yield different FWHM estimates, these figures should not be interpreted as a strict algorithm-agnostic ranking; rather, they indicate the achievable performance under each system’s typical workflow.

In comparison with the Inveon PET scanner (Siemens) [22], which uses 1.5 × 1.5 × 10 mm^3^ LYSO crystals—very close in size to the 1.457 × 1.457 × 12 mm^3^ crystals of PKU-PET-III—the reported spatial resolutions at the CFOV were 1.69 mm (tangential), 1.68 mm (radial), and 1.71 mm (axial) with OSEM reconstruction. PKU-PET-III achieved 1.0, 1.0, and 1.6 mm in the corresponding directions. Thus, despite nearly identical crystal pitch, PKU-PET-III demonstrated improved tangential and radial resolution, as well as comparable axial performance, suggesting that the detector geometry and readout design may help explain the observed improvement.

Across the entire measurement range, at both the axial center and a quarter axial offset, the axial and tangential spatial resolutions exhibited good uniformity, ranging from 0.9 mm to 1.63 mm (FWHM). Future work will incorporate controlled light-spread crystal designs for DOI correction, further improving resolution.

Sensitivity measurements reached 8.74% with an energy window of 200–750 keV, meeting high count-rate demands. Under comparable energy window conditions, PKU-PET-III exhibited relatively higher detection efficiency than several commonly used saPET systems, such as NanoPET/CT [23] (7.7%, axial FOV 9.48 cm, 250–750 keV), Inveon PET (7.4%, axial FOV 12.7 cm, 250–750 keV), and Metis PET/CT [24] (7.7%, axial FOV 122 mm, 200–750 keV). For comparison with our previous system, PKU-PET-II achieved a sensitivity of only 1.35% with a 300–700 keV window, representing more than a six-fold improvement in PKU-PET-III. Moreover, the peak sensitivities of PKU-PET-III for the 300–750 keV and 350–750 keV windows were 6.04% and 4.89%, respectively, illustrating the expected sensitivity–scatter trade-off. Overall, these results demonstrate that PKU-PET-III achieves a substantial gain in sensitivity under all tested conditions.

The use of a 200 keV lower threshold in early tests prioritized sensitivity and NECR characterization but comes at the cost of higher scatter acceptance. Our comparative analysis across three windows explicitly quantifies this trade-off; therefore, we recommend 300–750 keV as the default operating window for typical preclinical imaging with PKU-PET-III, reserving 200–750 keV for high count-rate assessments.

The mean energy resolution of 12.5% ensures effective scatter suppression and reliable quantitative performance. Compared to the Metis small-animal PET system [24] (produced by Madic Technology Co., Ltd., Linyi, Shandong, China), both systems feature an identical axial FOV of 122 mm. However, under the same 200–750 keV energy window, our system achieved a higher sensitivity (8.74%) than Metis (7.7%), which may be attributed to the smaller crystal thickness of 10 mm used in Metis, potentially limiting γ-photon detection efficiency [25]. Compared with PKU-PET-II (1.35%), the new system shows a substantial improvement, primarily attributed to the expansion of the axial FOV from 32 mm to 122 mm.

In Micro-Derenzo phantom imaging, Rods with diameters as small as 0.6 mm could be visualized, confirming the sub-millimeter resolution capability of the system. Such high resolution is particularly advantageous for mouse brain studies and tumor microenvironment imaging.

The count-rate and NECR measurements demonstrated that PKU-PET-III maintains stable performance across the activity range relevant for preclinical imaging studies. Within the investigated activity range, PKU-PET-III exhibited stable count-rate performance without signs of saturation. Since these activities already cover or exceed typical injected doses for small-animal imaging, the system is expected to provide reliable NECR and image quality in routine preclinical studies.

Overall, this PET system demonstrates high spatial resolution, sensitivity, and imaging uniformity, fulfilling diverse small-animal molecular imaging requirements. Its large transverse FOV and resolution performance make it well-suited for precise anatomical-functional correlation in preclinical research. With further advances in detector technology and reconstruction algorithms, this platform is poised to make significant contributions to drug development, pathology research, and integrated multimodal imaging.

## Figures and Tables

**Table 1:** System comparison of PKU-PET-II with PKU-PET-III.

	Parameters	PKU-PET-II	PKU-PET-III
Detector level	Crystal material	LYSO	LYSO
	No. of crystals per block	8 × 8	8 × 8
	Crystal size (length × width × thickness, mm^3^)	2.0 × 2.0 × 15.0	1.457 × 1.457 × 12
	Crystal rings	16	64
	Photodetector	SensL ArrayC-10035-64P	Hamamatsu S14160-4075HS
	No. of pixels per SiPM/MPPC array	8 × 8	4 × 4
	Size of SiPM/MPPC pixel (mm^2^)	2 × 2	3 × 3
System level	No. of detector modules	22	32
	No. of blocks per module	2	8
	Ring diameter (mm)	100	129
	Transaxial FOV (mm)	60	81
	Axial FOV (mm)	32	122
	Coincidence window (ns)	6	6
	Energy window (keV)	300–700	200–750/300-750
	Reconstruction algorithm	3D OSEM	3D OSEM

**Table 2:** Uniformity test result, including mean, maximum, and minimum values of activity concentration and %STD in the volume of interest (VOI).

	Mean (kBq/cc)	Maximum (kBq/cc)	Minimum (kBq/cc)	%STD
Uniformity	724.38	913.26	548.85	7.97

**Table 3:** SOR and %STD measured in the cold region.

	Mean of SOR	STD of SOR
Water	0.284	27.9%
Air	0.282	39.12%

## Data Availability

The data presented in this study are available on request from the corresponding author due to institutional and ethical restrictions.

## References

[1] Kitson S., Cuccurullo V., Ciarmiello A., Salvo D., Mansi L. (2009). Clinical applications of positron emission tomography (PET) imaging in medicine: Oncology, brain diseases and cardiology. Curr. Radiopharm..

[2] Galldiks N., Lohmann P., Albert N.L., Tonn J.C., Langen K.-J. (2019). Current status of PET imaging in neuro-oncology. Neuro-Oncology Adv..

[3] Anand S., Singh H., Dash A. (2009). Clinical applications of PET and PET-CT. Med. J. Armed Forces India.

[4] Schnöckel U., Hermann S., Stegger L., Law M., Kuhlmann M., Schober O., Schäfers K., Schäfers M. (2010). Small-animal PET: A promising, non-invasive tool in pre-clinical research. Eur. J. Pharm. Biopharm..

[5] Fine E.J., Herbst L., Jelicks L.A., Koba W., Theele D. (2014). Small-animal research imaging devices. in Seminars in nuclear medicine. Semin. Nucl. Med..

[6] Miyaoka R.S., Lehnert A.L. (2020). Small animal PET: A review of what we have done and where we are going. Phys. Med. Biol..

[7] Nanni C., Rubello D., Fanti S. (2007). Role of small animal PET for molecular imaging in pre-clinical studies. Eur. J. Nucl. Med..

[8] Yamamoto K., Yamamura K., Sato K., Ota T., Suzuki H., Ohsuka S. Development of multi-pixel photon counter (MPPC). Proceedings of the 2006 IEEE Nuclear Science Symposium Conference Record.

[9] Lecoq P., Gundacker S. (2021). SiPM applications in positron emission tomography: Toward ultimate PET time-of-flight resolution. Eur. Phys. J. Plus.

[10] Goertzen A.L., Van Elburg D. (2018). Performance characterization of MPPC modules for TOF-PET applications. IEEE Trans. Radiat. Plasma Med. Sci..

[11] Yu X., Zhang X., Zhang H., Peng H., Ren Q., Xu J., Peng Q., Xie S. (2022). Requirements of scintillation crystals with the development of PET scanners. Crystals.

[12] Freire M., Echegoyen S., Gonzalez-Montoro A., Sanchez F., Gonzalez A.J. (2022). Performance evaluation of side-by-side optically coupled monolithic LYSO crystals. Med. Phys..

[13] Cucarella N., Barrio J., Lamprou E., Valladares C., Benlloch J.M., Gonzalez A.J. (2021). Timing evaluation of a PET detector block based on semi-monolithic LYSO crystals. Med Phys..

[14] Hu P., Hua Z., Ma L., Qian S., Wu Q., Wang Z. (2022). Study on the optimized energy resolution of scintillator detectors based on SiPMs and LYSO: Ce. J. Instrum..

[15] Xie Z., Li S., Zhou K., Vuletic I., Meng X., Zhu S., Xu H., Yang K., Xu B., Zhang J. (2018). PKU-PET-II: A novel SiPM-based PET imaging system for small animals. Nucl. Instrum. Methods Phys. Res. Sect. A Accel. Spectrometers Detect. Assoc. Equip..

[16] NEMA (2008). Performance Measurements for Small Animal Positron Emission Tomographs.

[17] Cherry S.R., Sorenson J.A., Phelps M.E., ScienceDirect (Online service) (2003). Physics in Nuclear Medicine.

[18] Hamill J.J. 2D energy histograms for scatter estimation in an SiPM PET scanner. Proceedings of the 2019 IEEE Nuclear Science Symposium and Medical Imaging Conference (NSS/MIC).

[19] Cherry S.R., Shao Y., Silverman R., Meadors K., Siegel S., Chatziioannou A., Young J., Jones W., Moyers J., Newport D. (1997). MicroPET: A high resolution PET scanner for imaging small animals. IEEE Trans. Nucl. Sci..

[20] Doss K.K.M., Mion P.E., Kao Y.-C.J., Kuo T.-T., Chen J.-C. (2022). Performance evaluation of a PET of 7T Bruker Micro-PET/MR based on NEMA NU 4-2008 standards. Electronics.

[21] Farquhar T., Chatziioannou A., Cherry S. (2002). An evaluation of exact and approximate 3-D reconstruction algorithms for a high-resolution, small-animal PET scanner. IEEE Trans. Med. Imaging.

[22] Constantinescu C.C., Mukherjee J. (2009). Performance evaluation of an Inveon PET preclinical scanner. Phys. Med. Biol..

[23] Szanda I., Mackewn J., Patay G., Major P., Sunassee K., Mullen G.E., Nemeth G., Haemisch Y., Blower P.J., Marsden P.K. (2011). National Electrical Manufacturers Association NU-4 performance evaluation of the PET component of the NanoPET/CT preclinical PET/CT scanner. J. Nucl. Med..

[24] Liu Q., Li C., Liu J., Krish K., Fu X., Zhao J., Chen J.C. (2021). Performance evaluation of a small-animal PET/CT system based on NEMA NU 4–2008 standards. Med. Phys..

[25] Stickel J.R., Cherry S.R. (2004). High-resolution PET detector design: Modelling components of intrinsic spatial resolution. Phys. Med. Biol..

